# *Bacillus velezensis* NC-B4 as a promising antifungal agent for biocontrol of *Candida auris*

**DOI:** 10.3389/fcimb.2025.1515537

**Published:** 2025-09-02

**Authors:** Chunxi Yang, Chaoyu Cui, Yanru Chen, Zimei Peng

**Affiliations:** ^1^ Institute of Clinical Medicine, Jiangxi Provincial People’s Hospital, The First Affiliated Hospital of Nanchang Medical College, Nanchang, China; ^2^ Jiangxi Key Laboratory for Excavation and Utilization of Agricultural Microorganisms, Jiangxi Agricultural University, Nanchang, China

**Keywords:** antifungal activity, *Bacillus velezensis* NC-B4, biological control, *Candida auris*, genome-sequence analysis

## Abstract

**Introduction:**

*Candida auris*, known as the "super fungus", is commonly existed in hospital. The treatment of *C. auris* infection is difficult for its multi-drug resistance and difficult to accurately detect. The use of synthetic antibacterial agents has caused major problems such as drug-resistance and environment pollution and negatively affects non-target species. Microbial biocontrol agents (probiotics) are needed for fungal infection. *Bacillus* and related genera produce a variety of bioactive substances. As probiotics, it has been widely studied in the field of medicine and is a novel microbial factor for biological control.

**Methods:**

*B. velezensis* NC-B4 was isolated using gradient dilution method. Then it was identified by phylogenetic analysis and physiological and biochemical characteristics. The antibacterial mechanism of NC-B4 was explored by detecting cellulase, protease and genomic analysis. Then antimicrobial effects were analyzed by detecting the growth and biofilm of *C. auris* BJCA001. Finally, the cytotoxicity and the protective effect on mice were analyzed by cell line and mouse systemic infection models.

**Results:**

We isolated *B. velezensis* NC-B4, which showed cellulase, protease enzyme activity and antimicrobial effects against human pathogenic fungi by inhibiting the growth of Candida auris, *Cryptococcus neoformans, Candida albicans* and mycelial fungus. *B. velezensis* NC-B4 inhibited the biofilm formation and growth of C. auris. *B. velezensis* NC-B4 has a protective effect against the toxicity of Candida auris in A549 cell line and mouse systemic infection models. The complete genome of *B. velezensis* NC-B4 was 3.93 Mb with a 46.5% G+C content and possessed the macrolactin H, bacillaene, fengycin, difficidin, bacillibactin and bacilysin biosynthesis cluster, which known as key factors in biological control.

**Discussion:**

The results of the present study indicated that *B. velezensis* NC-B4 has antimicrobial properties for its cellulase, protease and antibacterial secondary metabolites, thereby inhibiting the growth of pathogenic bacteria and the formation of biofilms. *B. velezensis* NC-B4 is expected to be developed as a source for probiotics or new antibiotics.

## Introduction

*Candida auris*, an invasive fungal pathogen commonly colonized in skin, the respiratory tract, and urinary tract, has emerged globally as a multidrug-resistant fungal pathogen (Abdolrasouli et al., 2017; Welsh et al., 2017). There are six distinct clades of *C. auris* based on genetic and genomic information and locations of first isolates: Clade I (South Asian), Clade II (East Asian), Clade III (South African), Clade IV (South American), Clade V (Iran), and Clade VI (Singapore) (Bhargava, 2025). Ninety percent of clinical isolates of *C. auris* are resistant to fluconazole, and their sensitivity to other azole antifungal drugs, 5-fluorocytosine, amphotericin B, and echinocandins is also changeable, which often leads to blood infection with high mortality (Chowdhary et al., 2018). At present, antifungal drugs are still the main measures to treat *C. auris* infection, but the problems of drug resistance and environmental pollution caused by long-term use of antifungal drugs have attracted more and more attention (Osei Sekyere, 2018). Therefore, it is urgent to search for new antifungal methods to solve the drug resistance problem.

*Bacillus* is the most abundant group of bacteria in the rhizosphere of plants (Choudhary and Johri, 2009; Shao et al., 2022). The bioactive substances produced by *Bacillus* are harmless to livestock and poultry and can kill bacteria, some fungi, parasites, some viruses, and tumor cells, including drug-resistant strains, and are widely used in industry, agriculture, and medical production (Elshaghabee et al., 2017; Zalila-Kolsi et al., 2023). In addition, the microecological preparation prepared by *Bacillus* has played an important role in the treatment of intestinal flora imbalance, *candida* infection, and prevention of wound surface infection (Garvey et al., 2022; Zou et al., 2022; Ramesh and Roy, 2023; Kizhakkekalam, 2022). As a probiotic, it has been widely studied in the medical field and is an ideal new biological control microbial factor. For example, *Bacillus licheniformis* can inhibit *Staphylococcus*, *Candida albicans*, yeast, and *Escherichia coli*, and was made into capsules and oral liquids with living strains to treat intestinal diseases (Ramirez-Olea et al., 2022); the combination of intestinal ecological preparation of *Bifidobacterium* and *Bacillus licheniformis* and chemotherapy drugs cannot only kill and promote apoptosis of H22 ascites cancer cells but also prolong the life cycle of tumor mice and improve the effect of chemotherapy, which laid the foundation for clinical trials (Hirozawa et al., 2023).

There are some reports on the study of *B. velezensis* as a biological control microbial factor, the possible mechanisms of *B. velezensis* exert the antifungal effects are as follows: There are many genes responsible for the biosynthesis of antifungal compounds; it was reported that *B. velezensis* KTA01 can produce lipopeptide, which displayed prominent antifungal activity against *B. dothidea* KACC45481 (Kang et al., 2024). The research on the mechanism of *B. velezensis* HeN-7 CFS antifungal action demonstrated that HeN-7 CFS induced the membrane lipid peroxidation in *B. sorokiniana*, leading to the disruption of cell membrane integrity and resulting in the leakage of cell contents (Lin et al., 2024). *B. velezensis* CFS may inhibit *C. fioriniae* through interference with ribosomes, genetic information processing, cell membrane metabolism, and energy metabolism (Fu et al., 2024).

In this study, we aimed to screen isolates and identify functional characteristics of *B. velezensis* NC-B4 for developing biological control agents against *C. auris*, which is called super fungus. The antifungal effect of *B. velezensis* NC-B4 was detected against yeast and filamentous human pathogenic fungus by measuring the antifungal zone, then the protection of *B. velezensis* NC-B4 on mouse systemic infection with *C. auris* was detected by measuring the fungal burden [colony-forming unit (CFU)] in each organ after *C. auris* infection. We provide an understanding of the antibacterial mechanism of action of *B. velezensis* NC-B4 by detecting extracellular enzyme activity such as cellulase and protease and analyzing the secondary metabolite genes. We also evaluated the toxicity of *B. velezensis* NC-B4 by detecting the killing of NC-B4 on A549 cells. Based on the effective properties of NC-B4 for its antifungal action, we propose NC-B4 be suggested as a useful biological control agent (probiotic) for the medical and health industry.

## Materials and methods

### Strains and culture conditions

All the strains used in this study are listed in **Table 1**. Bacterial strains were grown at 35°C with shaking at 200 rpm in Lurai-Bertani broth (10 g tryptone, 5 g yeast extract, and 10 g NaCl in 1 L, pH 7.0). Fungi were grown at 28°C in Yeast Extract Peptone Dextrose Medium (10 g yeast extract, 20 g peptone, 20 g dextrose, and 20 g agar in 1 L). *Candida auris* BJCA001, *Cryptococcus neoformans* H99, *Candida albicans* SC5314, and four mycelial fungi were obtained from our laboratory. Bacterial growth was determined by measuring optical density at a wavelength of 600 nm.

**Table 1:** Bacterial and fungal strains used in this study.

Strain	Reference or Source
*B. velezensis* NC-B4	This study
*Candida auris* BJCA001	Laboratory collection
, *Cryptococcus neoformans* H99	Laboratory collection
*Candida albicans* SC5314	Laboratory collection
*Lasiodiplodia theobromae*	Laboratory collection
*Absidia*	Laboratory collection
*Cunninghamella bertholletiae*	Laboratory collection
*Trichophyton schoenleini*	Laboratory collection

### Isolation, screening, and identification of bacterial strains

*B. velezensis* NC-B4 was collected from plant rhizosphere soil (Nanchang, China) and isolated using the gradient dilution method. In detail, plant rhizosphere soil was collected from a hospital, park, and mountain and prepared into a 10% soil suspension (10^−1^), then the soil suspension was diluted by a 10-fold gradient (10^−2^, 10^−3^, 10^−4^, 10^−5^). Soil suspension (100 µl) of each concentration was spread on the LB plates, and the plates were incubated at 35°C until single colonies grew. Then pick up and inoculate the colonies on the YPD plates that contain *C. auris* BJCA001 (10^8^ CFU/ml). Finally, we selected the single colony that produced the inhibition zone for further identification. The detection method of physiological and biochemical characteristics refers to Burgey’s Manual of Determinative Bacteriology or the instruction of the kit (Hopebio, HBIG14). Molecular identification was performed by using primers (27F/1492R: AGAGTTTGATCCTGGCTCAG/AGAGTTTGATCCTGGCTCAG; gyrA-F/gyrA-R: CAGTCAGGAAATGCGTACGTCCTT/CAAGGTAATGCTCCAGGCATTGCT; rpoB-F/rpoB-R: AGGTCAACTAGTTCAGTATGGAC/AAGAACCGTAACCGGCAACTT) to amplify and sequence the fragments of 16S rRNA, *gyrA*, and *rpoB*, respectively (Lu et al., 2021).

### Phylogenetic and statistical analysis

For phylogenetic analysis, 16S rDNA, *gyrB*, and *rpoB* sequences closely related to our sequences were retrieved from GenBank based on BLAST results from the National Center for Biotechnology Information. Maximum likelihood (ML) phylogenies were constructed using the ML method in IQTREE v1.6.12 (http://iqtree.cibiv.univie.ac.at/). A bootstrap based on 1,000 replicates was analyzed, the confidence of the nodes was evaluated, and all parameters were kept at the default setting (Nguyen et al., 2015). The trees were visualized using FigureTree v1.4.3 and Adobe Illustrator CC 2018.

### Enzymatic activity analysis

Cellulase activity was evaluated by cellulase detection plate and DNS (di-nitrosalicylic acid) colorimetry methods. Cellulase detection plate method references (Shen et al., 2020) with minor changes: overnight culture was diluted to an OD600 of 0.01, and 2 µl bacterial suspensions were plotted in cellulase detection medium, then the plates were incubated at 35°C. After 48h, the plates were stained with 0.5% Congo red for 30 min and incubated with 1 M NaCl solution for 10 min at room temperature. Finally, the plates were washed three times by water, and cellulase activity in the plates was assessed by measuring the diameter of the degradation circle. Each treatment was replicated at least three times. The detailed steps of DNS colorimetry are as follows: *B. velezensis* NC-B4 isolate was grown in LB broth medium for 24h at 37 ± 2°C and then centrifuged at 13,000 rpm for 5 min. 0.5 ml of the supernatant (enzyme solution) was mixed with 1.5 ml of CMC-Na solubilized in phosphate buffer (1%) and incubated at 40°C for 30 min. 1.5 ml of dinitrosalicylic (DNS) acid reagent was added, and the mixture was boiled for 5 min; then cooled down and chilled to 25 ml, and the absorbancy was measured at 520 nm. One unit of enzyme activity was defined as 1 µmol glucose formed per minute.

Protease activity was evaluated by milk plate. In brief, we prepared an LB plate with 5% milk, then added NC-B4 fermentation broth supernatant (OD600 of 2.0, 3.0, 4.0) and incubated at 37°C for 24h. Protease activity was assessed by measuring the diameter of the degradation circle. Another method for analyzing the protease activity was determined by following previously published methods (Wang et al., 2021).

### Antifungal analysis

The antifungal ability of *B. velezensis* NC-B4 was evaluated using the disk diffusion method against several yeasts and filamentous fungi from clinical isolation. For yeast fungi, we prepared the YPD plate with *C. auris* BJCA001, and 10 µl of 1 × 10^6^ CFUs/ml of the culture suspension was distributed into the hole. After culturing at 35°C for 24h, the diameter of the inhibition zone was measured. For filamentous fungi, a pathogenic agar block was prepared and placed in three corners of the plate, and 10 µl of 1 × 10^6^ CFUs/ml of the culture suspension was distributed in the center. Then culturing at 28°C for 3–5 days, the antifungal activity of NC-B4 was assessed by determining the radial mycelial growth of the fungal pathogen.

### Cell growth analysis

For this assay, we firstly prepared NC-B4 fermentation broth supernatant (OD600 of 3.0), which was filtered by a 0.22 µm membrane. *C. auris* BJCA001 cells were incubated overnight (OD600 of 2.0) and then diluted 1,000 times using YPD medium. Then, 50 µl of diluted cell suspension containing NC-B4 fermentation broth supernatant (5, 10, 20 µl) were added to the 96-well plate in triplicate at 35°C for 2 days, then supplemented with YPD medium to 100 µl. We measured OD_600_ every six hours then plotted the growth curve.

### Biofilm formation assays

Biofilm formation was tested by determining the ability of fungal cells to adhere to the wells of 96-well polypropylene microtiter dishes. *C. auris* was grown overnight at 35°C and diluted to 1,000 times by using minimal medium (2 g glycerin, 2g mannitol, 10.5 g K_2_HPO_4_, 4.5 g KH_2_PO_4_, 2 g (NH_4_)_2_SO_4_, 0.2 g MgSO_4_·7H2O, 0.005 g FeSO_4_, 0.01 g CaCl_2_, 0.002 g MnCl_2_ in 1 L). Then *C. auris* suspension [with 0, 5, and 10 µl NC-B4 fermentation broth supernatant (OD600 of 3.0)] was added to 96-well polypropylene microtiter plates (100 μl per well) and incubated at 35°C with shaking at 200 rpm for 18h. To remove planktonic cells, we discarded the supernatant and washed twice and stained for 20 min with 1% (wt/vol) crystal violet. Then washed using water, added 200 μl ethanol to the well, and measured the absorbance at 595 nm (Huber et al., 2001).

### Cytotoxicity assays

Cytotoxicity was assessed by measuring the release of lactate dehydrogenase (LDH) from A549 cells. The 1 × 10^4^ A549 cells were routinely grown in Dulbecco’s Modified Eagle Medium (DMEM) supplemented with 1% fetal bovine serum (FBS) in a 96-well plate before infection. *C. auris* BJCA001 strain was grown in YPD medium at 35°C, then centrifuged and resuspended in DMEM medium (diluted to OD600 = 1), and different concentrations of NC-B4 fermentation broth supernatant (10%, 20%, and 40%) were added. A549 cells were infected with fungi or fungi with NC-B4 fermentation broth supernatant at 10^9^ CFU/ml for 8h. After the 8h incubation, culture supernatants were collected, and LDH in the supernatant was measured following the instruction of the LDH Cytotoxicity Assay Kit (Beyotime Biotechnology, China, C0016). Finally, the cytotoxicity was calculated relative to that of the uninfected control (Yang et al., 2017).

### Mouse systemic infection models analysis

All the animal experiments were approved by the Ethics Committee at the Jiangxi Provincial People’s Hospital (approval number KT2023-012). Male BALB/c mice (20–22 g) were used for fungal burden assays; five mice were used for each treated group [phosphate buffered saline (PBS) control, *C. auris* BJCA001, *C. auris* BJCA001 + 50% NC-B4 fermentation broth], and 2.5 × 10^7^ cells of BJCA001 in 250 µl PBS were injected into a mouse via tail vein. Mice were humanely killed at 48h after injection. Different organ tissues (liver, kidney, spleen, lung, and brain) of each infected mouse were removed, weighed, homogenized, and diluted in PBS for CFU calculation on YPD medium (Du et al., 2012; Xie et al., 2013).

### Whole genome sequencing and analysis

*B. velezensis* NC-B4 cells were incubated overnight and collected. Genomic DNA was extracted using a bacterial genome extraction kit (Beyotime Biotechnology, China, D0091) according to the manufacturer’s instructions. Whole-genome sequencing was performed using a combination of Illumina NovaSeq 6000 (Illumina, San Diego, CA, USA) and Nanopore PromethION platforms. For short reads sequencing on the NovaSeq 6000 platform, a small fragment library was prepared using the VAHTS^®^ Universal Plus DNA Library Prep Kit for MGI V2/for Illumina V2 (Vazyme, China) with an average insertion size of 300 bp. For long-read sequencing, the libraries were prepared using the SQK-LSK110 ligation kit and using the standard protocol.

The assembly of the genome was performed with Unicycler software (0.5.0). Then, Prodigal (v2.6.3), Aragorn (v1.2.38), RNAmmer (v1.2), and Infernal (v1.1) were used for predicting the coding genes, tRNA, rRNA, and mRNA genes, respectively. BLAST software was used for function annotations of genes against Cluster of Orthologous Groups of proteins (COG, http://blast.ncbi.nlm.nih.gov/Blast.cgi), and Kyoto Encyclopedia of Genes and Genomes (KEGG, https://www.kegg.jp/) databases.

## Results

### Screening and identification of bacterial strains

**Figure 1:**
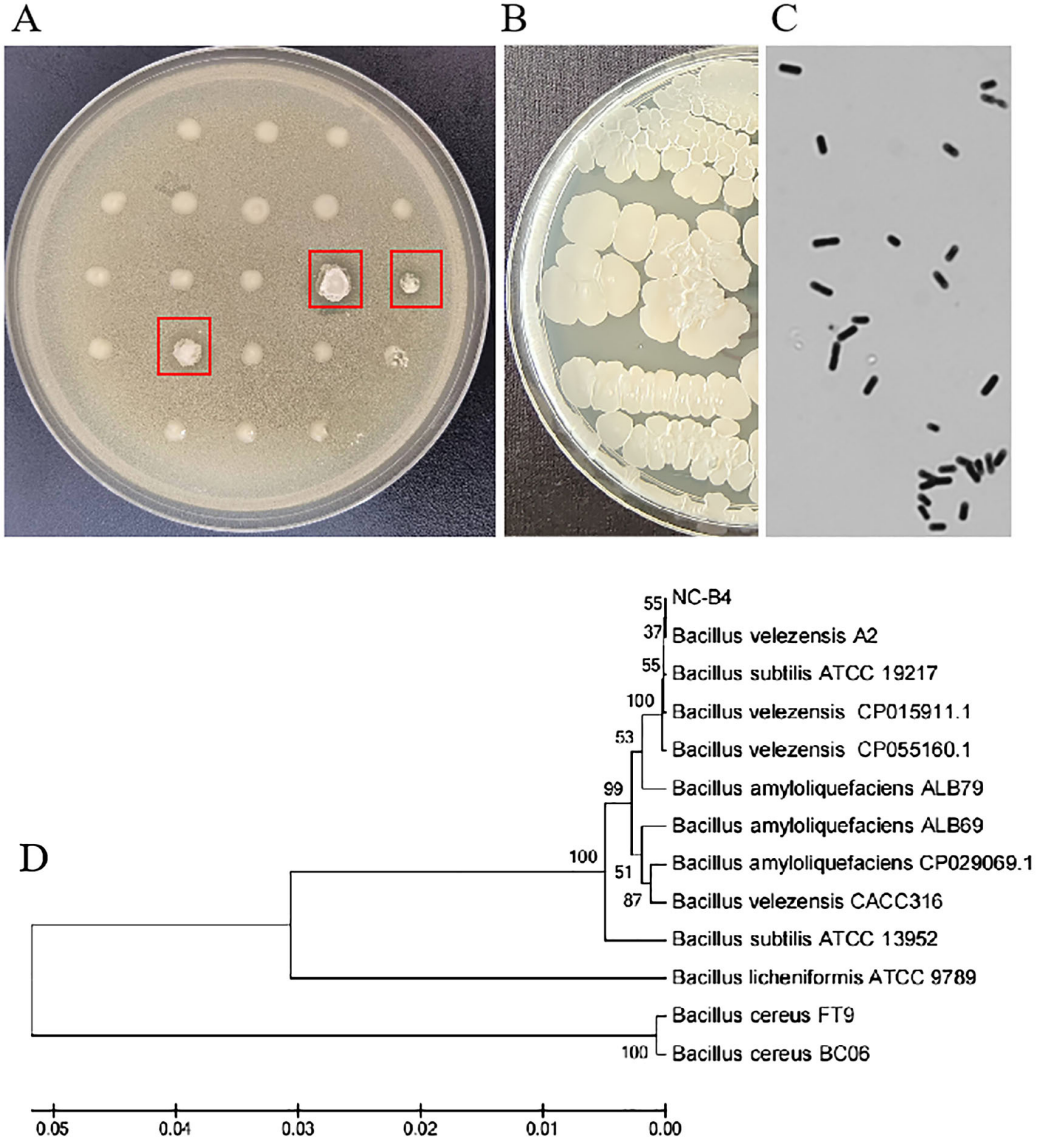
Colony morphology and phylogenetic tree. **(A)** Preliminary screening picture, strains which can inhibit the growth of *C. auris* were marked with a red arrow. **(B)** *B. velezensis* NC-B4 was inoculated on LB plate at 35°C for 24h, it could form round, milky white, opaque, with dry and wrinkled surface, irregular edge, and sunken center colonies. **(C)** Observation under microscope, the cells were rod-shaped, single or paired, the spore is nearly round, proximal, and the sporocystis are enlarged. **(D)** The phylogenetic tree, a maximum likelihood phylogenetic tree was established based on the concatenation of multiple sequences (16S rRNA, gyrA, and rpoB). In the phylogenetic tree, the NC-B4 and isolates of B velezensis clustered together with 100% bootstrap support.

To study the function of microbial biocontrol agents (probiotics) in fungal infection, we collected soil and prepared the soil suspension, then coated the plates. We selected the single clone and inoculated it on a plate that contained *C. auris* BJCA001. Then, screened strains that can inhibit the growth of *C. auris* were marked with a red arrow (**Figure 1A**). Then, we selected the number 4 strain with good antifungal effect; it could form round, milky white, opaque colonies, with dry and wrinkled surfaces, irregular edges, and sunken center on LB agar (**Figure 1B**). The cells were rod-shaped, single or paired, the spore is nearly round and proximal, and the sporocysts are enlarged (**Figure 1C**). It was identified as *B. velezensis* based on physiological and biochemical characteristics (**Table 2**) and molecular characteristics, including 16S rRNA, *gyrA*, and *rpoB* gene sequences, so we named it Nan chang *B. velezensis* 4(NC-B4). In order to further confirm its classification status, an ML phylogenetic tree was established based on the concatenation of multiple sequences (16S rRNA, *gyrA*, and *rpoB*). In the phylogenetic tree, the NC-B4 and isolates of *B. velezensis* clustered together with 100% bootstrap support (**Figure 1D**). Therefore, the NC-B4 was identified as *B. velezensis*.

**Table 2:** Physiological and biochemical characteristics of *B. velezensis* NC-B4.

Biolog	Result	Biolog	Result	Biolog	Result
Cell morphology	rod shape	D-glucose	+	tyrosine hydrolysis test	+
Gram stain	+	D-fructose	+	H2S production	+
Amylolysis	–	Sucrose	+	Methyl red test	–
catalase test	+	Maltose	+	Indole test	–
PH5.7 Broth	+	Dynamic test	+	urease	–
Nitrate reduction	+	Casein hydrolysis	+	oxidase	–
Gelatin liquefaction	+	Propionate utilization	–	Phenylalanine deaminase	–
L-arabinose	+	Citrate utilization	–	7% Nacl Salt tolerance test	+
D-xylose	+	V-P test	–	10% Nacl Salt tolerance test	+
D-mannitol	+	xylan	–	Lactose	+

+, positive; -, negative.

### Enzyme activity of *B. velezensis* NC-B4

**Figure 2:**
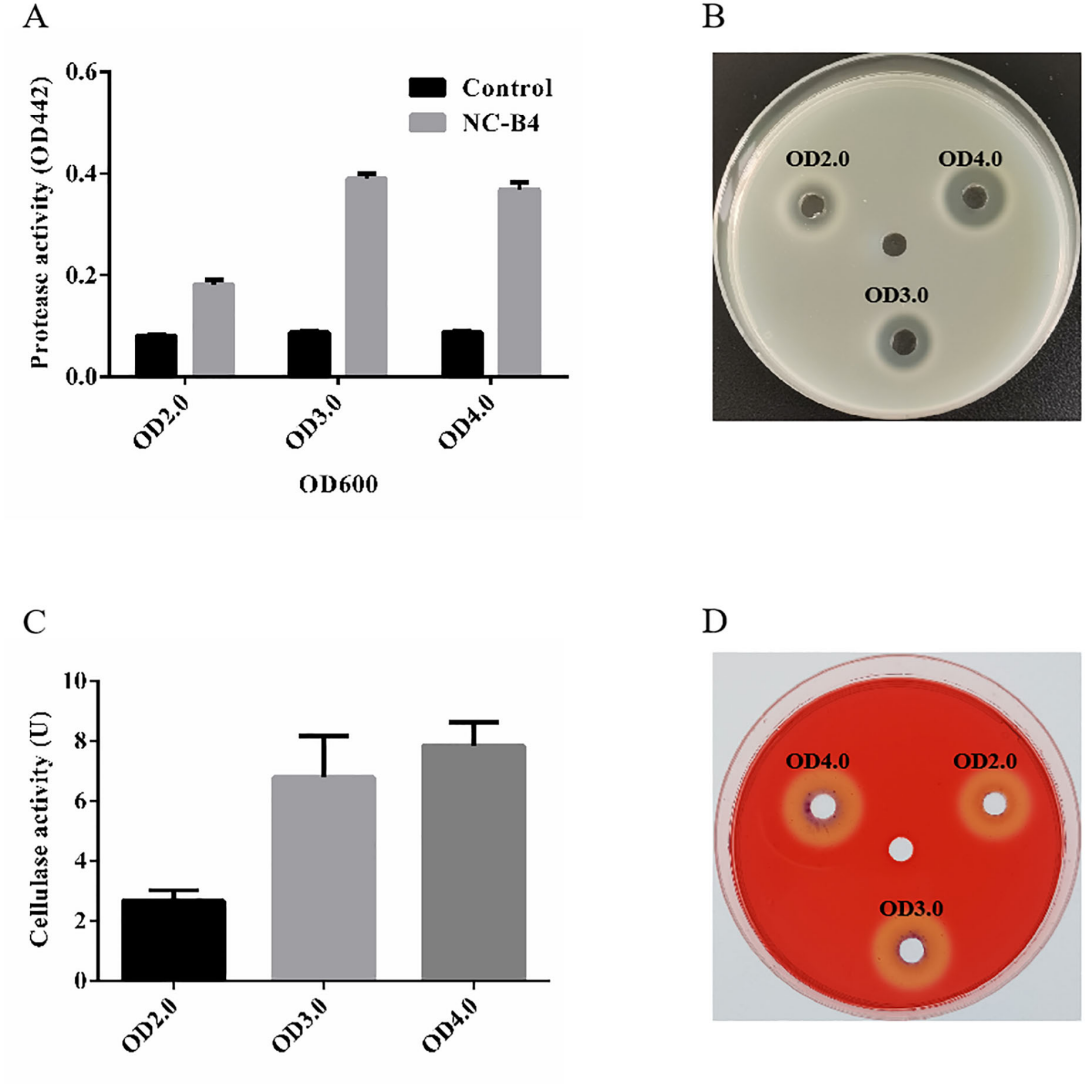
Enzyme activity of *B. velezensis* NC-B4. The protease **(A, B)** and cellulase **(C, D)** activities in fermentation broth were detected at different OD values (2.0, 3.0, and 4.0). **(A)** OD442 represents the strength of protease activity, NC-B4 has an obvious protease activity compared with clear LB medium (control) at different OD values. **(B)** NC-B4 fermentation broth can form a decomposition ring on the 5% milk plate, the larger the degradation ring, the stronger the protease activity. The cellulose activity of NC-B4 fermentation broth at OD2.0, 3.0, and 4.0. by absorbance method **(C)** and cellulase detection plate method **(D)**, the higher the enzyme activity value (U) and the larger the degradation circle are, and the stronger of cellulase activity will be. Each experiment was repeated three times.

To understand the antifungal mechanism of NC-B4, the extracellular enzyme activity of NC-B4 in different growth stages was detected by the plate method and the absorbance method. As shown in **Figure 2A**, NC-B4 has an obvious protease activity compared with clear LB medium, and the activity of protease increased with the increase of cell concentration and became stable after OD 600 reached 3.0. Another method (5% milk plates) reached the same conclusion (**Figure 2B**). Similarly, the measured cellulose activity of NC-B4 fermentation broth was 2.7U, 6.8U, and 7.8U at OD 600 2.0, 3.0, and 4.0, respectively, which showed a higher cellulose activity (**Figure 2C**). It also showed the same result by the cellulase detection plate method (**Figure 2D**).

### *In-vitro* antifungal effects against human pathogenic fungi

**Figure 3:**
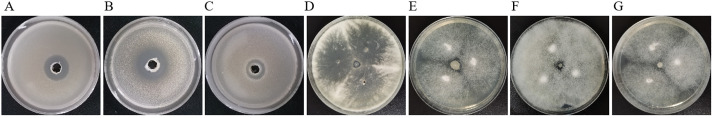
Antifungal activity of test strains against human pathogenic fungi. Inhibition effect of *C. auris* BJCA001 **(A)**, *C. neoformans* H99 **(B),** *C. albicans* SC5314 **(C)**, *L. theobromae* **(D)**, *Absidia* **(E)**, *C. bertholletiae* **(F)**, *T. schoenleini* **(G)**. Each experiment was repeated three times. The diameter of inhibitory zone represents antagonistic effects, NC-B4 strongly inhibited yeast fungi growth of *C. auris* BJCA001, Cryptococcus neoformans H99, *Candida albicans* SC5314, while weakly inhibited mycelial growth of *Lasiodiplodia theobromae, Absidia, Cunninghamella bertholletiae*, and *Trichophyton schoenleini*.

In order to detect whether *B. velezensis* NC-B4 has an antagonistic effect on other human pathogenic fungi, we selected yeast and filamentous fungi for the antagonistic activity assay. As shown in **Figure 3**, NC-B4 inhibited the growth of all seven human pathogenic fungi. However, the antagonistic effects on yeast and filamentous fungi were significantly different. NC-B4 strongly inhibited yeast fungi growth of *C. auris* BJCA001, *Cryptococcus neoformans* H99, and *Candida albicans* SC5314, while weakly inhibiting mycelial growth of *Lasiodiplodia theobromae*, *Absidia*, *Cunninghamella bertholletiae*, and *Trichophyton schoenleini*. The detailed antagonistic effect of strain NC-B4 on seven human pathogenic fungi was listed in **Table 3**; the diameter (mm) of the inhibitory zone or inhibition rate(%)represents antagonistic effects.

**Table 3:** Antagonistic effect of *B. velezensis* NC-B4 on 7 human pathogenic fungi.

Pathogens	Inhibitory zone diameter (mm)
*Candida auris* BJCA001	17.2 ± 0.91 mm
*Cryptococcus neoformans* H99	15.2 ± 0.85 mm
*Candida albicans* SC5314	8.1 ± 0.43 mm
*Cunninghamella bertholletiae*	4.1 ± 0.45 mm
*Trichophyton schoenleini*	4.1 ± 0.50 mm
*Absidia*	5.9 ± 0.65 mm
*Lasiodiplodia theobromae*	5.0 ± 0.75 mm

### *B. velezensis* NC-B4 inhibited the biofilm formation and growth of *C. auris*

**Figure 4:**
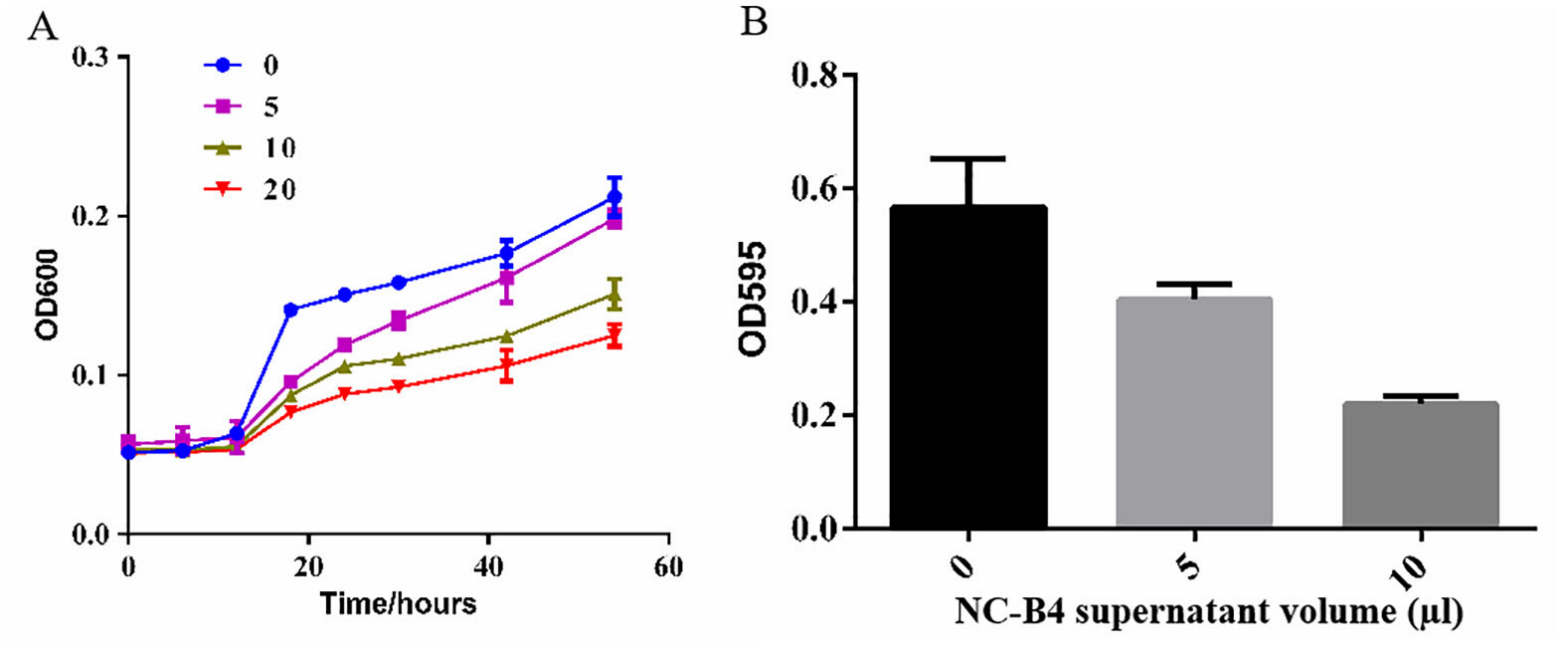
The supernatant *B. velezensis* NC-B4 controls the phenotypes in *C. auris*. Growth curve **(A)**, OD600 represents the density of strain, NC-B4 fermentation broth can inhabit the growth of *C. auris* when added 5, 10, and 20 µl of the supernatant in 100 µl at different cultivation periods, respectively; effect on *C. auris* biofilm formation **(B)**, OD595 represents the biofilm production, it was reduced when added 5, 10 µl of the supernatant in 100 µl, respectively. The data shown are the mean of three replicates, and error bars indicate the standard deviation. The experiment was repeated three times.

Biofilm is an important virulence factor, which is related to antibiotic resistance, escape of microbes from the body’s immune system, recalcitrant infections, and biofilm-associated deaths. So we evaluated the capacity of *B. velezensis* NC-B4 to inhibit the biofilm formation of *C. auris*. We obtained supernatant of NC-B4 fermentation broth then tested the effects on the growth curve and biofilm formation of *C. auris* BJCA001. We found NC-B4 fermentation broth can inhibit the growth of *C. auris* when added 5, 10, and 20 µl of the supernatant in 100 µl, respectively (**Figure 4A**). The biofilm production reduced to 67% and 33% when 5 and 10 µl of the supernatant were to 100 µl, respectively (**Figure 4B**).

### The effects of *B. velezensis* NC-B4 fermentation broth on the pathogenicity of *C. auris*

**Figure 5:**
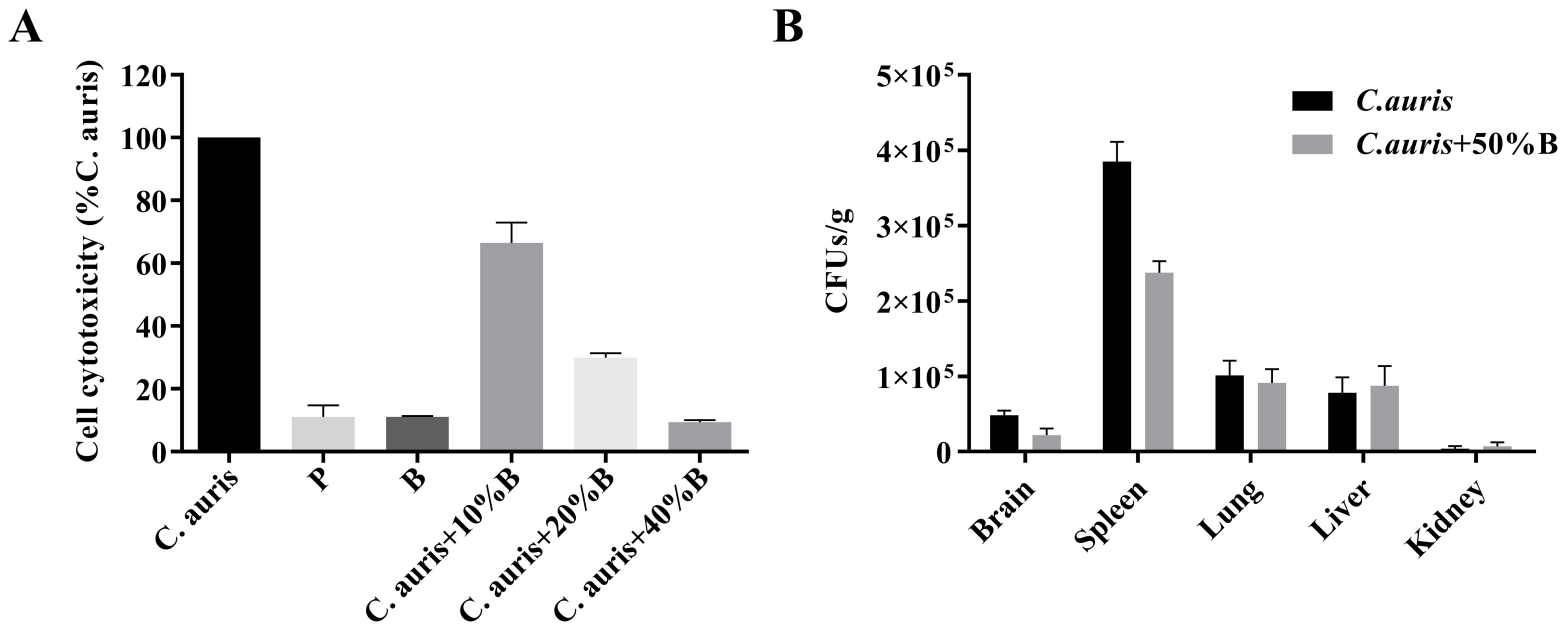
The effects of *B. velezensis* NC-B4 extraction on the pathogenicity of *C. auris* were determined using the A549 cell line **(A)** and mouse model **(B)**. They were repeated three times. **(A)** Cell cytotoxicity was detected and measured as LDH release. P and B represent adding 40 µl of NC-B4 pellet (1 × 10^9^ CFU/ml) and NC-B4 supernatant. LDH released by *C. auris* BJCA001 was arbitrarily defined as 100% and used to normalize the LDH release ratios of *B. velezensis* NC-B4 fermentation broth and the BJCA001 with different gradient fermentation broth. The data shown are the mean of three replicates, and error bars indicate the standard deviations. **(B)** Measurement of the fungus colony-forming units (CFUs) in different tissues of mouse after 48h of infection, it revealed that the addition of 125 µl/250 µl of NC-B4 supernatant (50% B) decreased the fungal burden (CFU) in spleen and brain, while there are no significant difference in kidney, lung, and liver.

To detect the effects of fermentation broth on the cytotoxicity of *C. auris*, we measured the cytotoxicity by quantifying the release of LDH into the supernatant of a human cell line, A549. The result showed that the NC-B4 pellet (labeled P in **Figure 5A**) and supernatant (labeled B in **Figure 5A**) did not exhibit toxicity when added at 40 µl/100 µl; however exogenous addition of the NC-B4 supernatant at 10, 20, and 40 µl/100 µl (10%, 20%, and 40% B) reduced *C. auris* virulence by 33%, 70%, and 90%, respectively (**Figure 5A**). NC-B4 supernatant also reduced *C. auris* infection in the mouse systemic infection models. Measurement of the fungus CFUs in different tissues of the mouse revealed that the addition of 125 µl/250 µl of NC-B4 supernatant decreased the fungal burden (CFU) in the spleen and brain, while there was no significant difference in the kidney, lung, and liver (**Figure 5B**).

### Genome feature of *B. velezensis* NC-B4

**Figure 6:**
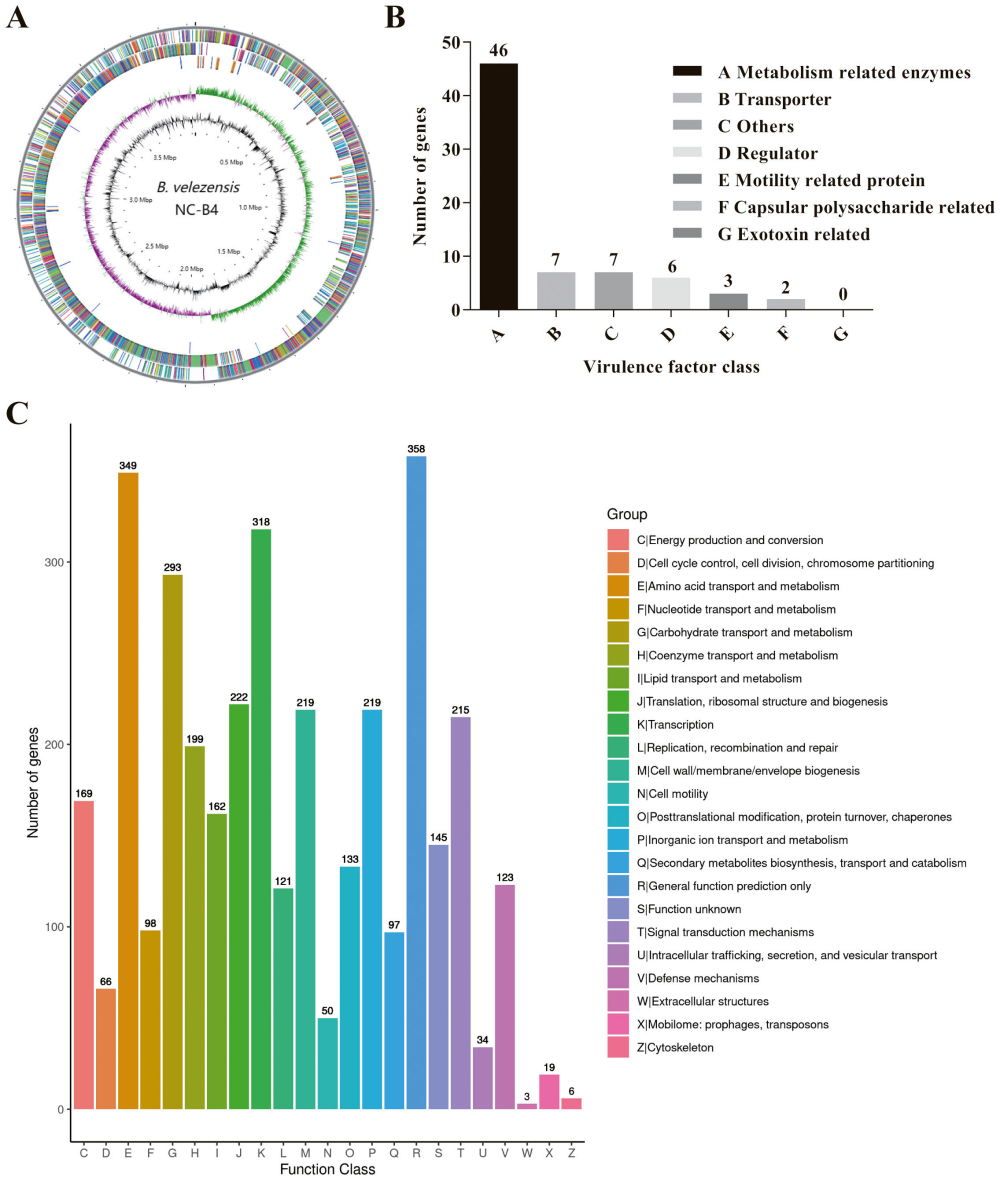
Genome Feature of *B. velezensis* NC-B4. Circular genome maps of *B. velezensis* NC-B4 chromosome **(A)**. Circles from the outside to the center denote rRNA and tRNA gene, reverse strand coding sequence, forward strand coding sequence, GC skew, and GC content. Genome number of virulence factor clusters of orthologous groups category **(B)**. Compared with virulence Factors Database (VFDB), 71 genes with pident and Qcovs values greater than 50 were obtained and classed. Most of them belong to metabolism related enzymes, some of them are transporter, regulator, motility related proteins, there is no exotoxin related genes. Genome number of clusters of orthologous groups category **(C)**. Among these coding sequences, 3,618 genes were distributed to 23 orthologous clusters, more than 250 genes were classified into functional categories for general function prediction only (*n* = 358), amino acid transport and metabolism (*n* = 349), transcription (*n* = 318), carbohydrate transport and metabolism (*n* = 293).

The whole genome of NC-B4 contains a 3,929,792 base pair circular chromosome with a 46.5% G+C content. The genome contains 3,747 protein-coding sequences (CDSs), 27 rRNAs, and 86 tRNAs (**Figure 6A** and **Table 4**). Among these coding sequences, 3,618 genes were distributed to 23 orthologous clusters (**Figure 6C**). More than 250 genes were classified into functional categories for general function prediction only (*n* = 358), amino acid transport and metabolism (*n* = 349), transcription (*n* = 318), and carbohydrate transport and metabolism (*n* = 293) (**Figure 6C**). To assess the safety of NC-B4, we analyzed the virulence factors in the genome data of the strain NC-B4. Compared with the virulence factors database (VFDB), 71 genes with pident and Qcovs values greater than 50 were obtained and classed. Most of them belong to metabolism-related enzymes; some of them are transporters, regulators, or motility-related proteins; and there are no exotoxin-related genes (**Figure 6B** and **Table 5**). The gene clusters related to secondary metabolite clusters identified in the genome of *B. velezensis* NC-B4 are listed in **Table 6**. In detail, NC-B4 possesses six metabolite clusters, five of them have 100% similarity, which are conserved in all *B. velezensis* members; the remaining one has 82% similarity. This group of six metabolite clusters comprises macrolactin H, bacillaene, fengycin, difficidin, and bacillibactin gene clusters, encoding the antibacterial or antifungal bioactivity (**Table 6**). The whole genome sequences of the NC-B4 isolate have been deposited in GenBank (Bioproject number: PRJNA995027).

**Table 4:** The whole genome overview of *B. velezensis* NC-B4.

Chromosome	
Assembly Size (bp)	3929792
DNA G+C Content (%GC)	46.5
CDSs	3747
rRNAs	27
tRNAs	86

**Table 5:** Virulence factor classes identified in the genome of *B. velezensis* NC-B4.

SeqID	Pident	Qcovs	Class	Annotation
ctg_03427	93.9	100	Metabolism related enzymes	CapC, involved in Poly-gamma-glutamate synthesis
ctg_03428	93.13	100	Metabolism related enzymes	CapB, involved in Poly-gamma-glutamate synthesis
ctg_03737	78.4	100	Metabolism related enzymes	UDP-glucose 4-epimerase GalE
ctg_03009	72.1	100	Metabolism related enzymes	isochorismatase, DhbB
ctg_03237	71.0	100	Metabolism related enzymes	phosphopyruvate hydratase
ctg_02287	70.4	100	Metabolism related enzymes	NADP-dependent phosphogluconate dehydrogenase
ctg_03012	69.7	100	Metabolism related enzymes	2,3-dihydroxybenzoate-2,3-dehydrogenase, DhbA
ctg_01050	64.0	100	Metabolism related enzymes	lipoate protein ligase
ctg_03506	62.6	100	Metabolism related enzymes	urease beta subunit UreB, urea amidohydrolase
ctg_01916	84.4	99	Metabolism related enzymes	gamma-glutamyltranspeptidase, required for polyglutamate anchoring to peptidoglycan
ctg_03010	81.1	99	Metabolism related enzymes	2,3-dihydroxybenzoate adenylase DhbE
ctg_03406	78.8	99	Metabolism related enzymes	UTP--glucose-1-phosphate uridylyltransferase GalU
ctg_03008	70.3	99	Metabolism related enzymes	non-ribosomal peptide synthetase, DhbF
ctg_03011	65.3	99	Metabolism related enzymes	isochorismate synthase DhbC
ctg_01893	64.0	99	Metabolism related enzymes	UTP--glucose-1-phosphate uridylyltransferase
ctg_01398	62.1	99	Metabolism related enzymes	ATP-dependent protease
ctg_03569	59.3	99	Metabolism related enzymes	nitrate reductase subunit beta
ctg_01581	58.3	99	Metabolism related enzymes	signal peptidase II
ctg_01224	57.2	99	Metabolism related enzymes	UDP-glucose 4-epimerase GalE
ctg_03509	56.2	99	Metabolism related enzymes	urease alpha subunit UreA
ctg_03241	55.2	99	Metabolism related enzymes	type I glyceraldehyde-3-phosphate dehydrogenase
ctg_02509	54.8	99	Metabolism related enzymes	mannose-6-phosphate isomerase, class I
ctg_03282	52.7	99	Metabolism related enzymes	CpsD/CapB family tyrosine-protein kinase
ctg_03415	52.4	99	Metabolism related enzymes	mannose-6-phosphate isomerase, class I
ctg_02481	52.0	99	Metabolism related enzymes	aspartate/glutamate racemase family protein
ctg_03470	51.3	99	Metabolism related enzymes	CpsD/CapB family tyrosine-protein kinase
ctg_03570	50.6	99	Metabolism related enzymes	nitrate reductase subunit alpha
ctg_00876	50.4	99	Metabolism related enzymes	glutamate-1-semialdehyde 2,1-aminomutase
ctg_01631	50.0	99	Metabolism related enzymes	short chain dehydrogenase/reductase family oxidoreductase
ctg_03468	60.0	98	Metabolism related enzymes	UDP-glucose/GDP-mannose dehydrogenase family protein
ctg_03280	57.2	98	Metabolism related enzymes	polysaccharide biosynthesis protein
ctg_02617	56.5	98	Metabolism related enzymes	glutamate-1-semialdehyde 2,1-aminomutase
ctg_00912	55.9	98	Metabolism related enzymes	catalase
ctg_03712	55.6	98	Metabolism related enzymes	mannose-6-phosphate isomerase, class I
ctg_03271	63.6	97	Metabolism related enzymes	sugar transferase
ctg_03300	77.9	96	Metabolism related enzymes	ATP-dependent Clp protease proteolytic subunit
ctg_03339	60.0	96	Metabolism related enzymes	prolipoprotein diacylglyceryl transferase
ctg_03269	60.7	95	Metabolism related enzymes	aminotransferase class I/II-fold pyridoxal phosphate-dependent enzyme
ctg_02406	52.1	95	Metabolism related enzymes	superoxide dismutase
ctg_03508	50.0	94	Metabolism related enzymes	urease alpha subunit UreA
ctg_01601	50.0	92	Metabolism related enzymes	adenylyl-sulfate kinase
ctg_01694	61.4	89	Metabolism related enzymes	undecaprenyl diphosphate synthase
ctg_02121	60.2	89	Metabolism related enzymes	aspartate 1-decarboxylase
ctg_02047	56.6	89	Metabolism related enzymes	trifunctional thioredoxin/methionine sulfoxide reductase A/B protein
ctg_02157	58.0	88	Metabolism related enzymes	nucleoside-diphosphate kinase
ctg_03178	50.0	81	Metabolism related enzymes	copper-translocating P-type ATPase
ctg_03426	78.9	99	transporter	CapA, required for Poly-gamma-glutamate transport
ctg_00409	53.2	99	transporter	iron chelate ABC transporter ATP-binding protein VctC
ctg_03732	51.8	98	transporter	sn-glycerol-3-phosphate ABC transporter ATP-binding protein UgpC
ctg_03066	50.7	98	transporter	sn-glycerol-3-phosphate ABC transporter ATP-binding protein UgpC
ctg_03150	50.2	96	transporter	ABC transporter ATP-binding protein
ctg_03115	65.3	95	transporter	ABC transporter ATP-binding protein
ctg_01632	63.0	95	transporter	acyl carrier protein
ctg_03469	51.4	100	others	hypothetical protein
ctg_02056	80.7	100	others	hemolysin III family protein
ctg_00117	78.4	99	others	endopeptidase Clp ATP-binding chain C
ctg_01605	52.2	99	others	fibronectin-binding protein
ctg_00642	74.9	96	others	chaperonin GroEL
ctg_02451	61.6	93	others	chaperone protein DnaK
ctg_03007	56.3	90	others	MbtH-like protein
ctg_00145	74.4	99	regulator	elongation factor Tu
ctg_01674	66.1	98	regulator	response regulator
ctg_00235	50.2	98	regulator	two-component system response regulator VirR
ctg_01839	52.6	97	regulator	chemotaxis response regulator CheY
ctg_02423	62.4	73	regulator	RNA polymerase sigma factor
ctg_03376	50.0	73	regulator	carbon storage regulator CsrA
ctg_01676	53.6	95	molity related protein	flagellar biosynthesis protein FliP
ctg_01396	50.0	93	molity related protein	flagellar motor protein MotB
ctg_01665	55.7	94	molity related protein	flagellar protein export ATPase FliI
ctg_03471	55.0	89	capsular polysaccharide related protein	capsular polysaccharide biosynthesis protein
ctg_03405	62.1	95	capsular polysaccharide related protein	capsular polysaccharide biosynthesis protein Cps4I

**Table 6:** Secondary metabolite clusters identified in the genome of *B. velezensis* NC-B4.

Most similar known cluster	Synthetase Type	Genes	Size (Kb)	Bioactivity	Similarity
macrolactin H	transAT-PKS	lacC,defB,pksE_1,fadA_1,pksN_1,acpP_1,acpP_2,acpP_3,rutD_1,yfeW,pdhA,pdhB,pdhD_2,speA_2,suhB	87.8	antibacterial	82%
bacillaene	NRPS,T3PKS,transAT-PKS	pbpX,tdh_1,miaB,baeB,baeC,baeD,baeE,acpK,pksG_1,pksH,pksI_1,pksJ_1,pksL,pksM,pksN_2,pksR,pksS,aprX	100.6	multiple	100%
fengycin	NRPS,betalactone,transAT-PKS	yjmD,uxuB,lgrD,dltA_1,dltA_2,fenF,bdhA_1,bioI,bioB_1,bioF,bioK,gtaB_1,crt,yngG,cfiB,fadD3,bcd,ppsE,ppsD,ppsC,ppsB,ppsA,ggt,adh_2	134.3	Antifungal	100%
difficidin	transAT-PKS	gloB_3,pksI_2,pksG_2,yjiB_2,tdh_2,acpP_4,fadA_2,pksJ_2,fadA_3,fabG_3,menE_1,acpP_5,pksE_2,namA,rutD_3,gndA,pepT_1,pccB,artP	93.8	multiple	100%
bacillibactin	NRPS,RiPP-like	rutB_2,yueD,mbtH,dhbF,dhbB,dhbE,dhbC,dhbA,besA,yumC,rimJ,ydfG	51.8	antibacterial	100%
bacilysin	other	yhdG_3,bacG,bacF_2,bacD,bacC_1,rocC_2,rocA,rfbC,rmlD,rfbB,rmlA	41.4	antibacterial	100%

## Discussion

*Bacillus* spp. have been widely studied as microbial biocontrol agents. As a useful microorganism in the medical industry, microecological preparations prepared by *Bacillus* play an important role in the treatment of intestinal flora disorders, *candida* infection, prevention of wound infection, and other medical processes (Garvey et al., 2022; Zou et al., 2022). *B. licheniformis* has an inhibitory effect on *Staphylococcus*, *Candida albicans*, *yeast*, and *Escherichia coli*; capsules and oral liquid made from *B. licheniformis* strains can treat intestinal diseases (Ramirez-Olea et al., 2022). In addition, intestinal ecological preparation of the combination of *bifidobacterium* and *bacillus licheniformis* combined with chemotherapy drugs cannot only kill and promote apoptosis of H22 ascites cancer cells but also prolong the life cycle of tumor mice and improve the effect of chemotherapy (H. S. et al., 2023). Here, we isolated and identified a *B. velezensis* NC-B4 (**Figure 1**) that has antifungal activity (**Figure 3** and **Table 3**).

Cell-wall lytic enzymes exert antifungal effects by destroying the fungal cell wall, cytoplasmic membrane or affect fungal growth and differentiation (Aimanianda et al., 2017; Choudhary et al., 2014). For example, cellulase enzymes degrade cell walls by cleaving the β-1,4-D-glycosidic bonds that connect the glucose units containing cellulose (Harrison and Bonning, 2010), and proteolytic enzymes capable of hydrolyzing polysaccharides adversely affect fungal growth and differentiation by dissolving or disturbing polymers in the cell wall of pathogenic fungi (Hasan et al., 2013). Here, we detected the potent activity of cellulase and protease in NC-B4 (**Figure 2**) that supports its correlation with the growth inhibition of various human pathogenic fungi (**Figure 3**). It has been reported that the enzymes produced by *Bacillus* spp., such as amylase, cellulase, and protease, are highly associated with antifungal activity against *Fusarium oxysporum* pathogens (El-Sersawy et al., 2021). Moreover, the synergistic effect of cell-wall lytic enzymes and secondary metabolites may enhance the antifungal effect (Kim et al., 2022).

*C. auris* is an emerging fungal pathogen that is becoming a serious global health threat. Due to its multidrug-resistant features, invasive infections of *C. auris* often results in high mortality rates (Du et al., 2020; Spivak and Hanson, 2018). Here, we showed that the antagonistic activity of NC-B4 on human pathogenic fungi revealed a broad capacity to inhibit the growth of fungus, especially on *C. auris* (**Figure 3** and **Table 3**). Biofilm is an important virulence factor that is related to fungal drug resistance (Rather et al., 2021). Culture broth of NC-B4 not only inhibited the growth and biofilm formation of *C. auris* (**Figure 4**) but also can reduce the cytotoxicity of *C. auris* to A549 cells (**Figure 5A**). In the mouse systemic infection model, treatment with culture broth of NC-B4 significantly decreased the fungal burden (CFU) in the spleen and brain (**Figure 5B**). For a good microbial biocontrol agent (probiotic), in addition to the function of inhibiting pathogens, the safety of the probiotic strain is very important. Here, we verified that NC-B4 has no toxicity to A549 cells (**Figure 5A**). We also analyze virulence factors in the genome data of the strain NC-B4; there are no toxin-related genes (**Figure 6B**).

Through whole genome sequencing and analysis, we predicted the genes and gene clusters involved in secondary metabolites produced by NC-B4. *Bacillus* spp. can produce multiple antimicrobials with a variety of chemical structures, including surfactin, fengycin, macrolactin H, bacillaene, difficidin, bacillibactin, bacilysin, and plantazolicin; they have different effects in the medical industry (Salazar et al., 2023; Sansinenea and Ortiz, 2011). In NC-B4, it exhibits a high genetic capacity for synthesizing cyclic lipopeptides (i.e., fengycin, bacillibactin) and polyketides (i.e., macrolactin H, bacillaene, and difficidin) (**Table 6**). Among them, the biosynthetic gene clusters of macrolactin H, bacillibactin, and bacilysin that have antibacterial activity were detected in the NC-B4 genome and had size of 87.8 kb, 51.8 kb, and 41.4 kb, respectively (**Table 4**). In addition, the gene cluster bae and pks associated with the biosynthesis of bacillaene were predicted in the NC-B4 genome (100.6 kb), bacillaene is known as a broad-spectrum antibiotic that inhibits bacterial protein. The gene cluster encoding fengycin synthesis was detected with a size of 134.3 kb, which has an antifungal function.

Numerous studies have been conducted to determine the impact of *Bacillus* secondary metabolite on pathogens (Fira et al., 2018; Hirozawa et al., 2023). It is noteworthy that on average around 5% of the whole genome of the *Bacillus* spp. is dedicated to the synthesis of secondary metabolites (Salazar et al., 2023). *B. subtilis* pB2-L produced plipastatin (the fengycin family), which inhibits *F. oxysporum* mycelium growth (Gao et al., 2017). *B. amyloliquefaciens* S76–3 produced plipastatin A and iturin A, which have clear antagonistic effects on *F. graminearum* (Gong et al., 2015). *B. velezensis* produces secondary metabolites and enzymes such as protease, chitinase, cellulase, and glucanase, and it inhibits *B. cinerea* growth, and so on (Fazle and Baek, 2020). However, it has only been reported that *Bacillus* can inhibit plant pathogenic fungi, and its inhibitory effect on human pathogenic fungi has not been reported (Fira et al., 2018). Here, we found *B.velezensis* NC-B4 also produces a variety of secondary metabolites and enzymes such as protease and cellulase, which have significant antagonistic effects on human pathogenic fungi, especially on *C. auris*. The whole genome analysis of secondary metabolites and the detection of enzymes provide scientific evidence of the effectiveness of NC-B4 as a biocontrol agent.

## Data Availability

The datasets presented in this study can be found in online repositories. The names of the repository/repositories and accession number(s) can be found in the article/supplementary material.
